# Evaluation of Target Attainment for Tobramycin in Children and Adults with Cystic Fibrosis

**DOI:** 10.3390/jcm13092641

**Published:** 2024-04-30

**Authors:** Sheseira T. L. Struiken, Danique Lobée, Eline L. van Tuinen, Daniel J. Touw, Hester van der Vaart, Arno R. Bourgonje, Bart L. Rottier, Gerard H. Koppelman, Paola Mian

**Affiliations:** 1 Department of Clinical Pharmacy and Pharmacology, University Medical Center Groningen, University of Groningen, 9712 GZ Groningen, The Netherlands; s.t.l.struiken@umcg.nl (S.T.L.S.); danique.lobee@hotmail.com (D.L.); elinevantuinen@hotmail.com (E.L.v.T.); d.j.touw@umcg.nl (D.J.T.); 2 Department of Pharmaceutical Analysis, Groningen Research Institute for Pharmacy, University of Groningen, 9712 GZ Groningen, The Netherlands; 3 Department of Pulmonary Diseases and Tuberculosis, University Medical Center Groningen, University of Groningen, 9712 GZ Groningen, The Netherlands; h.van.der.vaart@umcg.nl; 4 Department of Gastroenterology and Hepatology, University Medical Center Groningen, University of Groningen, 9712 GZ Groningen, The Netherlands; a.r.bourgjonje@umcg.nl; 5 Henry D. Janowitz Division of Gastroenterology, Department of Medicine, Icahn School of Medicine at Mount Sinai, New York, NY 10029, USA; 6 Department of Pediatric Pulmonology and Pediatric Allergology, University Medical Center Groningen, Beatrix Children’s Hospital, University of Groningen, 9712 GZ Groningen, The Netherlands; b.l.rottier@umcg.nl (B.L.R.); g.h.koppelman@umcg.nl (G.H.K.); 7 Department of Pediatric Pulmonology, University Medical Center Groningen, University of Groningen, Groningen Research Institute for Asthma and COPD (GRIAC), 9712 GZ Groningen, The Netherlands

**Keywords:** tobramycin, pharmacokinetics, cystic fibrosis, therapeutic drug monitoring

## Abstract

**Introduction:** Patients with cystic fibrosis (CF) commonly experience pulmonary exacerbations, and it is recommended by the TOPIC study to treat this with tobramycin at a dose of 10 mg/kg once daily. The aim of this study was to evaluate the target attainment of the current dosing regimen. **Methods:** A single-center retrospective cohort study of child and adult patients with CF who received tobramycin between 2019 and 2022 was conducted. Descriptive statistics and linear mixed models were used to assess target attainment for tobramycin. **Results:** In total, 25 patients (53 courses), of which 10 were children (12 courses) and 15 were adults (41 courses), were included. Those 25 patients all received 10 mg/kg/day. The tobramycin peak concentrations were supratherapeutic in 82.9% and therapeutic in 100.0% of adults and children, respectively. The trough concentrations were outside the target range in 0% and 5.1% of children and adults, respectively. We found lower tobramycin concentrations with the same dose in children compared to adults. **Conclusions:** This study illustrates the need to validate dosing advice in a real-world setting, as supratherapeutic concentrations of tobramycin were prevalent in adults with CF.

## 1. Introduction

People with cystic fibrosis (CF) are especially prone to developing endobronchial infections caused by a wide scala of bacteria. Bacteria commonly infecting the lungs include *Staphylococcus aureus*, *Haemophilus influenzae*, *Stenotrophomonas matophilia* and *Pseudomonas aeruginosa* [1]. Particularly, *Pseudomonas aeruginosa* is associated with progressive structural lung damage and accelerated lung function decline [2]. Pulmonary exacerbations caused by *Pseudomonas aeruginosa* in cystic fibrosis (CF) are usually treated with a beta-lactam antibiotic and tobramycin intravenously [3]. The Cystic Fibrosis Foundation Pulmonary Guidelines recommend opting for the once-daily administration of tobramycin intravenously three times per day for pulmonary exacerbations to optimize its effectiveness and reduce the likelihood of safety concerns such as nephrotoxicity [3,4]. The usual initial dosage of tobramycin in CF patients suggested by thes guidelines is 10 mg/kg/day [3,4,5,6] although the literature supports dosing ranging from 6 to 15 mg/kg/day [7,8,9,10,11,12,13]. Tobramycin has a narrow therapeutic window, and its antibacterial effect depends on the ratio of the maximal concentration (C_max_) to the Minimum Inhibitory Concentration (MIC) of the bacteria [14,15]. The target concentrations, according to the Dutch guidelines, are a peak of 25–30 mg/L and a trough of <0.5 mg/L [16]. However, it has to be noted that there is no consensus on the target concentrations in the different guidelines and clinical trials [4,6,14,17]. However, too-high trough concentrations are associated with increased risk of nephrotoxicity and ototoxicity [18]. Therapeutic drug monitoring (TDM) is recommended to optimize the dosage to achieve individual target concentrations [3,19]. The ability to reach effective and safe target concentrations with this currently used dosing regimen and the above-mentioned target concentrations is unclear and under investigation using real-world data. The aim of this study is to evaluate the target attainment of tobramycin at a dose of 10 mg/kg/day in child and adult CF patients.

## 2. Materials and Methods

### 2.1. Study Design, and Inclusion and Exclusion Criteria

This was a single-center retrospective descriptive study. Child and adult CF patients admitted to the University Medical Center Groningen (UMCG) for whom the first tobramycin concentration (trough, mid, peak) was determined as part of TDM after at least one dose of intravenous tobramycin were included. Per course, we included only the first steady-state concentration for tobramycin, before TDM alterations in dose or dose interval had occurred, as concentrations determined after TDM would not reflect the target attainment of the current dose. For tobramycin, a steady state was defined as three times the half-life of tobramycin, which is 1.4–3 h for children and 2.5–4 h for adults [14]. All measured samples were included. Doses and analytical results were fed into population pharmacokinetic software (MWPharm++, version 2.21 (Mediware, Prague, Czech Republic)) based on the population pharmacokinetic model of Touw et al. [15]. In addition, individual pharmacokinetics with peak and trough levels at steady state were calculated. These calculated values were used for evaluation. Patients were excluded if they (1) received a dose that was not concordant with the 10 mg/kg/day dosing regimen (which was defined as a daily drug dose and dose interval that deviated by >10% from the 10 mg/kg/day dosing advice) or, (2) received tobramycin inhalation therapy or tobramycin containing Selective Digestive Decontamination (SDD), (3) underwent extracorporeal membrane oxygenation (ECMO) or any form of renal replacement therapy during antibiotic treatment or (4) had no accurate (steady-state) trough, mid or peak concentration determined as part of TDM advice. Informed consent was waived by the UMCG medical ethics committee (METc 2022/609).

### 2.2. Data Collection

Clinical data were retrospectively collected from electronic health records from 1 October 2019 to 1 October 2022 and comprised demographics, admission data, antibiotic doses, dates and times of administration, tobramycin plasma concentrations and their date and time of determination, and serum creatinine concentrations. Drug concentrations were analyzed using EMIT (Enzyme Multiplied Immunoassay Technique) (Architect, Abbott Laboratories, Lake Bluf, IL, USA) for tobramycin. This assay has lower and upper limits of quantification of 0.2 and 40 mg/L for tobramycin. Data cleaning was carried out by the first authors of the manuscript (S.T.L.S., D.L. and E.L.v.T.) and all data extraction was double checked by the last author of the manuscript (P.M.). 

### 2.3. Data Analysis

The tobramycin courses were divided into three subgroups (subtherapeutic, therapeutic and supratherapeutic). The target concentrations were a peak of 25–30 mg/L one hour after start of administration, and trough of <0.5 mg/L 30 min before a new administration [14]. When the timing of samples deviated from the defined times, a one-compartment linear pharmacokinetic model was used to calculate peak and trough concentrations with MwPharm++ [17].

The differences in tobramycin peak and trough concentrations among patients treated with 10 mg/kg once daily (administered in 30 min) were additionally analyzed using linear mixed model analysis in order to confirm the observed differences in target attainment while accounting for the correlated nature of the data (i.e., some individuals having multiple concentration measurements). Age group (adults vs. children) was entered as a fixed effect, while a random effect was allowed for subject ID to study differences in the estimated concentrations. Akaike’s information criterion (AIC) was used to prioritize the best fitting covariance structure. Data analysis was performed using the SPSS Statistics software package (v. 28, SPSS Inc., Chicago, IL, USA).

## 3. Results

### 3.1. Patient Characteristics 

Screening retrieved 88 adults and children with CF who used tobramycin. Of these, 41 were excluded due to receiving either tobramycin inhalation or tobramycin containing SDD. Seven patients were excluded due to the absence of tobramycin concentration measurements, and four because of ECMO, dialysis or missing pharmacokinetic analysis of the concentration measurements. Furthermore, among the children in three (20.0%) of the courses, a dose different from 10 mg/kg was given. In all such cases, the doses were higher than 11 mg/kg. In one of these cases, it was explicitly stated that the higher dose was based on the effect of previous treatments. For the two other cases, there was no explanation. None of the doses were given in the emergency department. Among adults, in 43.2% of the courses, a dose different from 10 mg/kg was given. In 97.4%, the dose was less than 9 mg/kg. In 2.6%, the dose was higher than 11 mg/kg. In 42.1%, the dose was based on the effect of previous treatment. In the other cases, 95.5% received a non-CF dose of 7–8 mg/kg (6–9 mg/kg), with 23.8% given at the emergency department and 4.8% in the intensive care unit. There was no explanation for one case in which a dose of 4 mg/kg was given. The tobramycin target attainment was based on the group that received a dose of 10 mg/kg/day (with a margin of 1 mg/kg), leaving 10 children (12 courses) and 15 adults (41 courses) for data analysis (Table 1). 

**Figure 1:**
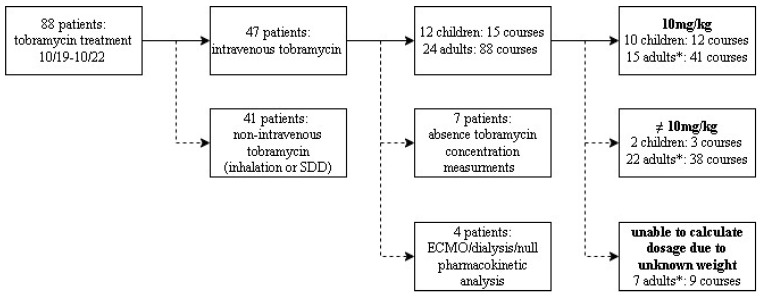
Flowchart indicating the total number of cycles, number of exclusions, reason for exclusion, total number of patients and tobramycin treatment cycles included, and stratification among the child and adult cohorts. * indicates that one patient could have multiple cycles consisting of both 10 mg/kg or ≠10 mg/kg. Those adults are counted in both groups.

In total, 1–2 samples per child or 1–8 samples per adult were obtained. The screening process is illustrated in Figure 1. 

### 3.2. Tobramycin Target Attainment in Children

**Figure 2:**
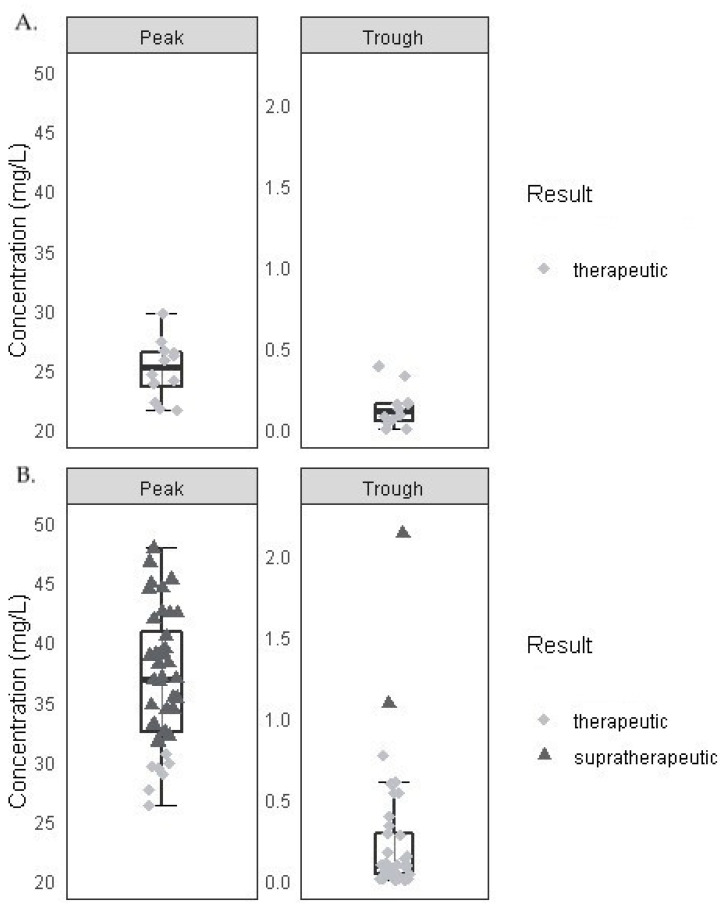
Concentrations of tobramycin in children with CF (**A**) and adults with CF (**B**) (supported by Table 2) after 10 mg/kg intravenous tobramycin.

The percentage of target attainment is presented in Figure 2A and Table 2. The median (IQR) tobramycin trough concentration was 0.11 (0.11) mg/L. The median (IQR) tobramycin peak concentration was 25.2 (2.91) mg/L, with 100% showing adequate concentrations. In 26.7% of the courses, creatinine was determined either in the 5 days before or after the start of treatment. 

Peak concentrations are defined as subtherapeutic if the concentration was below 20 mg/L, as therapeutic if the concentration was between 20 and 30 mg/L or as supratherapeutic if the concentration was above 30 mg/L. Trough concentrations are defined as therapeutic if the concentrations were <1.0 mg/L and supratherapeutic if the concentrations were >1.0 mg/L.

### 3.3. Tobramycin Target Attainment in Adults 

The percentage of target attainment is presented in Figure 2B and Table 2. The median (IQR) tobramycin trough concentration was 0.09 (0.25) mg/L, with 94.9% and 5.1% of the courses showing adequate and too-high concentrations, respectively. The median (IQR) tobramycin peak concentration was 36.9 (9.40) mg/L, with 17.1% and 82.9% of the courses showing adequate and too-high concentrations, respectively. In 28% of the courses, creatinine was measured at the start of treatment. In 25 out of 88 courses, creatinine was determined within the window of 5 days before and 5 days after the start of treatment.

### 3.4. Analysis of Tobramycin Peak and Trough Concentrations

Adults with CF demonstrated significantly higher estimated tobramycin peak concentrations compared to children with CF (*p* < 0.001, estimated marginal mean difference 12.12 mg/L), confirming the higher frequency of supratherapeutic concentrations in adults, as observed in the target attainment data. There was no significant difference in estimated tobramycin trough concentrations between adults and children with CF (*p* = 0.391), although lower estimated concentrations were observed among children compared to adults (estimated marginal mean difference: 0.11 mg/L).

## 4. Discussion

We retrospectively investigated tobramycin target attainment in children and adults with CF. Adequate trough concentrations, a marker for safety, were achieved with the current start dosing regimen of 10 mg/kg/day. Adequate peak concentrations, as a marker of efficacy, was reached for children in 100% of cases. Data from the adults showed that 82.9% had supratherapeutic peak concentrations and 0% had subtherapeutic concentrations. The safety of such high peak concentrations has not been established. Achievement of the target concentration is of utmost importance, as this is associated with optimal treatment, while suboptimal treatment can result in resistance or toxicity. Adults also showed larger interindividual variability, expressed as an IQR of 9.40 mg/L, compared with children (IQR of 2.91 mg/L). Since the peak concentration is mainly determined by the volume of distribution, the current dosing regimen of 10 mg/kg per day seems to overestimate the volume of distribution in adults, shown by the large proportion of too-high tobramycin peak concentrations. This is opposite to our tobramycin data for children, where adequate tobramycin peak concentrations were observed. From a pharmacological point of view and given that tobramycin is a hydrophilic drug, it is to be expected that children receiving the same dose as adults will attain lower tobramycin peak concentrations, as in general, children have relatively more body water than adults [18,19,20,21,22]. Tobramycin will primarily distribute into the hydrophilic compartments, such as blood and the extracellular volume. This is supported by the observed age-dependent trend of a decreasing volume of distribution with increasing age (Supplementary Figure S1) and has been described in the literature as well [8]. Overall, our results indicate that more target attainment is achieved in children compared to adults. This is not in line with previous published studies. For example, Touw et al. suggested that a 20% higher mg/kg dose is needed to achieve the same target concentrations for children as in adults [23]. The authors concluded that no significant difference occurs in clearance between children and adults with CF [23]. The retrospective chart review from Imburgia et al. analyzed 326 encounters and reported that only 43.5% achieved an adequate target concentration with dosages between 9.5 and 11.9 mg/kg/day [24]. The authors also concluded that increasing age was associated with higher initial target attainment and fewer dose modifications. There is an indication that younger children may require higher weight-based dosing to meet the defined target [24]. Vandenbussche and Homnick performed a prospective study in which 25 CF patients (14 children, 11 adults) received 10 mg/kg/day IV tobramycin, showing that only 42% of the CF patients obtained adequate peak (20–30 mg/L) concentrations [25]. Contradictory to our results, this study showed no differences between PK parameters in children and adults [25]. A hypothesis for the differences between our study and previously published studies could relate to the fact that the nutritional status in patients (both adults and children) with CF is improved. This significantly results in increased fat mass in both adults and children, which, for tobramycin, results in a decreased volume of distribution and thereby higher peak concentrations. Therefore, no good comparison can be made with previously published studies due to evolution in the CF field. 

It has to be noted that the target concentrations used in this study are based on the Dutch TDM guidelines [16]. However, to date, there is no consensus on the target concentrations in the different guidelines [4,6,17]. For example, the CF UK Trust Antibiotics Report of 2009 does not mention a target peak for tobramycin [6]. Other references and guidelines suggest different targets that would have a major impact on our results. The TOPIC study defined a peak target of 20 to 30 mg/L and a trough of <1 mg/L. The CFF guidelines recommend a peak of 25 to 35 mg/L [3] and an undetectable trough, and the Cystic Fibrosis Canadian guidelines suggest a peak of 20–40 mg/L and a trough <1 mg/L [17]. Within all the specified guidelines above, no level of evidence (grade) is provided for the specific target concentrations. 

Besides demonstrating the target attainment of tobramycin in both children and adults with CF, this study demonstrates that an initial dose of 10 mg/kg/day, according to the TOPIC study [4], is not consistently used in children and adults. The largest deviation from the 10 mg/kg/day dose is observed in adults, which is mostly due to the fact that when adults are admitted with pulmonary exacerbation, clinicians apply non-CF dosing of 5–7 mg/kg in 12.2% and 46.3% of cases in the ER and outside the ER, respectively. According to us, this is an alarming finding as, apparently, physicians in the ER are not aware that in CF, a higher initial dose is required, or that for tobramycin dosing, the starting dose as prescribed upon previous admission is used. It has to be noted that a limitation of this study is that this is a monocenter study. This indicates that the results from our study cannot be directly extrapolated to other centers. However, we want to point out that these issues can occur. Another limitation of this study is that the Minimum Inhibitory Concentrations (MIC) were not available and therefore could not be presented. Finally, another limitation of our study is that patients receiving ECMO or dialysis were excluded from our cohort. This limits the generalizability of our results. However, it should be noted that higher-quality tobramycin concentration time data (before and after the start of the extracorporeal technique) in more patients should be collected to investigate the impact of those extracorporeal techniques on the target attainment of tobramycin in patients with CF. 

Our data underline the necessity to describe additional covariates needed to explain interpatient variability. It should be noted that one of those covariates acting as a predictor of tobramycin elimination in CF patients is creatinine clearance [26]. From our study, it can be concluded that in clinical practice, creatinine concentrations are not frequently measured within an adequate time frame from tobramycin concentrations. Measuring creatinine serum concentration in an early stage prior to or simultaneously with measuring tobramycin concentration is required to enable early dose adjustments. Finally, the importance of CF-specific dosing regimens should also be emphasized in the ER context. 

It can be concluded that there is a need to re-evaluate the initial tobramycin dose based on present real-world data, as overall supratherapeutic peak tobramycin concentrations were prevalent in children and adults with CF, respectively. Over the last few decades, tremendous efforts have been made to develop, based on PKPD data, model-informed precision dosing (MIPD) [15,27,28,29]. These PKPD models have resulted in, for example, an initial dose of tobramycin of 15 to 17.5 mg/kg/day in children [26]. For adults, a decrease in the volume of distribution has been reported [15] and it seems logical that a lower dosage is needed. This is supported by our data (Supplementary Figure S1). Although these PKPD models have shown that different starting MIPDs are needed, these models can also be used to perform Bayesian simulations from the obtained TDM data to individualize dosing regimens further throughout individual patients’ therapy [5]. 

Further research should focus on performing a pooled analysis of tobramycin in children and adults with CF. In this way, a heterogeneous dataset will be created, covering all globally published PKPD data of tobramycin in CF patients. 

## 5. Conclusions

In conclusion, this study illustrates the need to validate dosing advice in a real-world setting as supratherapeutic concentrations of tobramycin were prevalent, mainly in adults with CF.

## Figures and Tables

**Table 1:** Clinical characteristics of the children and adults with CF. Data are presented as medians and interquartile ranges (IQRs) or numbers with percentages.

Characteristics	Children	Adults
**Total cohort**		
Total patients	12	24
Total courses	15	88
**10 mg/kg/day cohort**		
Total patients with 10 mg/kg/day	10	15
Total courses with 10 mg/kg/day	12	41
% male/female	50/50	53/47
Age	12 (5.25)	23 (4)
Weight	36.35 (19.85)	54.80 (6.6)
Dose (mg/kg/day)	10.07 (0.33)	10.0 (0.7)
Peak concentration (mg/L)	25.20 (2.91)	36.91 (9.40)
Trough concentrations (mg/L)	0.11 (0.11)	0.09 (0.25)
Creatinine level (μmol/L)	45.50 (8.5)	67.0 (35.5)

**Table 2:** Overview of the efficacy and safety target attainment of tobramycin in each cohort (supported by Figure 1).

	Peak Concentrations			Trough Concentrations	
Cohort	Subtherapeutic	Therapeutic	Supratherapeutic	Therapeutic	Supratherapeutic
Children	0% (n = 0)	100.0% (n = 12)	0% (n = 0)	100% (n = 12)	0% (n = 0)
Adults	0% (n = 0)	17.1% (n = 7)	82.9% (n = 34)	94.9% (n = 37)	5.1% (n = 2)

## Data Availability

The data are available upon reasonable request by contacting the corresponding author.

## Supplementary Materials

The following supporting information can be downloaded at: https://www.mdpi.com/article/10.3390/jcm13092641/s1, Figure S1: Volume of distribution of tobramycin in patients at various points in the human age span receiving 10 mg/kg/day.
