# Supplementary material for: Evaluation of Target Attainment for Tobramycin in Children and Adults with Cystic Fibrosis
Source: J Clin Med. 2024 Apr 30;13(9):2641. doi: 10.3390/jcm13092641 (PMC11084493; doi:10.3390/jcm13092641)

Supplemental Material

Supplemental Figure S1. Volume of distribution of tobramycin over the human-age span receiving 10 mg/kg/day.

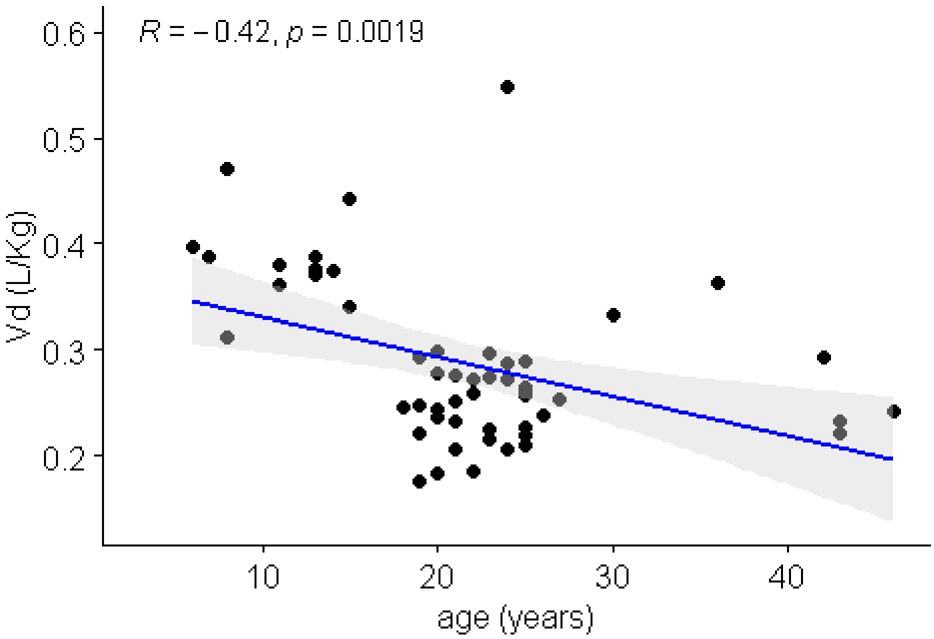

Supplement: Supplementary file 1 [file jcm-13-02641-s001.zip › jcm-2881453-supplementary.pdf]
